# Characteristics and driving mechanisms of species beta diversity in desert plant communities

**DOI:** 10.1371/journal.pone.0245249

**Published:** 2021-01-11

**Authors:** Lamei Jiang, Guanghui Lv, Yanming Gong, Yan Li, Hengfang Wang, Deyan Wu

**Affiliations:** 1 College of Resources and Environment Science, Xinjiang University, Urumqi, China; 2 CAS Key Laboratory of Biogeography and Bioresources in Arid Land, Xinjiang University, Urumqi, Xinjiang, China; Tennessee State University, UNITED STATES

## Abstract

Species dissimilarity (beta diversity) primarily reflects the spatio–temporal changes in the species composition of a plant community. The correlations between β diversity and environmental factors and spatial distance can be used to explain the magnitudes of environmental filtering and dispersal. However, little is known about the relative roles and importance of neutral and niche-related factors in the assemblage of plant communities with different life forms in deserts. We found that in desert ecosystems, the β diversity of herbaceous plants was the highest, followed by that of shrubs and trees. The changes in the β diversity of herbs and shrubs had stronger correlations with the environment, indicating that community aggregation was strongly affected by niche processes. The soil water content and salt content were the key environmental factors affecting species distributions of the herb and shrub layers, respectively. Spatial distance explained a larger amount of the variation in tree composition, indicating that dispersal limitation was the main factor affecting the construction of the tree layer community. The results suggest that different life forms may determine the association between organisms and the environment. These findings suggest that the spatial patterns of plant community species in the Ebinur Lake desert ecosystem are the result of the combined effects of environmental filtering and dispersal limitation.

## Introduction

Community assembly is important to the coexistence of species and the maintenance of biodiversity and is one of the hottest issues in contemporary community ecology [1–5]. For more than a century, ecologists have been trying to clearly explain how community assembly maintains biodiversity, with niche theory and neutral theory being two outstanding conceptual frameworks. Whether stochastic processes or deterministic processes are more important in community assembly and whether their relative contributions are stable are questions that have attracted much scholarly attention over time. However, the relative contributions of each type of process will change according to different scales, species groups, and ecosystem types [5–7]. Therefore, in order to understand the mechanisms of community assembly more accurately, it is necessary to consider the roles of multiple factors in the discussion of their effects.

Beta diversity indicates changes in community composition in space or time [8, 9] and provides an objective perspective for understanding the process of species aggregation within a community. At present, niche processes and neutral processes that affect beta diversity are largely individually regulated by environmental filtering or dispersal limitation [10–12]. The niche process is defined as a species adapting to a particular environment, thus generating a niche. Niches mean that environmental gradients have a filtering effect on species distribution. Essentially, the beta diversity of two communities or regions is the result of the environmental differences between them. In other words, communities with similar environments usually have similar species compositions, and the spatial differences in species composition will become larger with increasing environmental differences [11, 13, 14]. The niche process includes two levels: biological and abiotic interactions. Environment filtering (abiotic interactions) describes the environment as a metaphorical sieve that only permits species with particular traits or phenotypes to establish and persist in the absence of biotic interactions [15]. Here, we pay attention to environmental filtering because such processes determine the potential combination of species in the community, and biotic interaction operates in turn. The impact of these interactions can vary considerably with both extrinsic (e.g., environmental) and intrinsic (e.g., density) factors [16–18]. The diffusion process means that the beta diversity depends on the degree of isolation between communities or regions and the diffusion abilities of biological groups: the lower the isolation, the lower the beta diversity [19–21]. It is generally believed that geographical distance is the most important measurement of species diffusion limitation [12, 22]. Due to dispersal limitation, the similarity of community species composition should decrease with an increase in geographic distance [12, 22]. Hotspots in biodiversity can host studies that test the relative importance of environmental filtering and dispersal limitation processes in driving the formation of beta diversity in different biological groups.

The heterogeneity of plant community composition is driven by the distribution of soil resources and dispersal limitation, and research on these topics is relatively extensive. For example, random diffusion determines the richness and composition of species in the broad-leaved forest communities in Gutian Mountain, China [23]. Environmental factors limit the coverage of different functional groups in secondary forest communities in Pennsylvania and have an effect on community assembly [24]. Unlike tropical forest assembly, which is affected by dispersal limitation, temperate forest community assembly is affected by environmental filtering [25]. The assembly mechanism of temperate forest communities in Japan is greatly influenced by habitat. Mori and colleagues found that the assembly of high-altitude communities is a deterministic process, while the assembly of low-altitude communities is a random process [26]. The interaction of the soil phosphorus concentration, clay content, and fallow duration had a significant impact on the change in species composition in the Amazon River Basin [27]. In the arid areas of tropical Costa Rica, Werden and colleagues found that the distribution of most tree species was related to soil environmental factors [28], but soil factors alone did not explain the distribution of forest species in Indonesia [29]. Different ecosystems have different levels of sensitivity and tolerance to environmental factors. For tropical rainforests, light is the main factor affecting community structure, while for forests in arid areas, water is the main driving factor [30]. Therefore, different terrestrial ecosystems are affected by environmental filtering and dispersal limitation to varying degrees.

Arid regions (including semi-arid regions) account for approximately 20–25% of the global land area, and they are one of the most vulnerable ecosystems and a precious resource of global biodiversity. Arid ecosystems are one of the key areas used for globally significant research on biodiversity [31, 32]. Aridity is of great significance for the maintenance of desert plant community diversity and for explaining changes in diversity. Aridity acts as a strong environmental filter in drylands [33], affecting the plant community aggregation and controlling trait values and phylogenetic structures [15, 34, 35]. Increasing aridity may favor species with a diminished specific leaf area [36]. It also determines the type of root system, favoring roots more able to maximize water and nutrient acquisition during short peaks of resource availability [37]. Arid ecosystems are spatially heterogeneous and lack soil nutrients. Seed dispersal is quite limited in such harsh environments where adaptations for long-distance dispersal are rare and poor dispersibility is common [38]. It can be seen that both environmental filtering and dispersal limitation significantly affect the distribution and composition of desert plant species. Therefore, it is important to explore their relative effects on desert plant communities to deepen our understanding of desert ecosystem functions and thereby to aid in the protection and restoration of biodiversity.

Ebinur Lake wetland in Xinjiang Ebinur Lake Basin Wetland National Nature Reserve is located in China’s famous Alashankou wind channel and is a desert wetland ecosystem that is extremely unstable, with high sensitivity and vulnerability. The soil water and salt contents along the bank of the Aqikesu River are relatively high and decrease with distance from the river channel [39]. Previous studies have demonstrated that the distribution and replacement of plant species in the arid areas show a certain response pattern related to the distance from the river channel [40]. Therefore, studies on the pattern of desert plant diversity and its influential mechanisms in the Ebinur Lake Nature Reserve should be carried out in a direction perpendicular to the Aqikesu River, with the objective of answering the following questions: (1) What are the beta diversity characteristics of communities of different plant types in the desert ecosystem of Ebinur Lake Basin? (2) How can environmental filtering and diffusion processes explain the assembly of living plant communities? (3) Does the plant community assembly in the desert ecosystem conform to either niche theory or neutral theory?

## Materials and methods

### Overview of the research area

The Ebinur Lake National Wetland Nature Reserve, located in the northwest of Jinghe County, in the Xinjiang Uygur Autonomous Region (82 36’–83 50’ E,44 30’–45 09’ N), is the lowest depression and water and salt reservoir in the western Junggar Basin. This region has a typical temperate continental arid climate [41], with an annual precipitation of approximately 100 mm, evaporation that exceeds 1600 mm, approximately 2800 hours of sunshine, an extreme maximum temperature of 44°C, an extreme minimum temperature of -33°C, and an annual average temperature of 6–8°C [42]. The plant communities are mainly xerophytic and hyper-xerophytic desert species accompanied by a variety of halophytes, psammophytes, and aquatic species. The plant communities are dominated by small trees, shrubs, and subshrubs. The herbaceous plants are mainly perennial herbaceous plants, with ephemeral plants distributed in some of the desert areas. The study area is our field observation and research station for desert vegetation, and no permission is required to enter the field site to perform field vegetation surveys.

### Field survey and data generation

**Fig 1:**
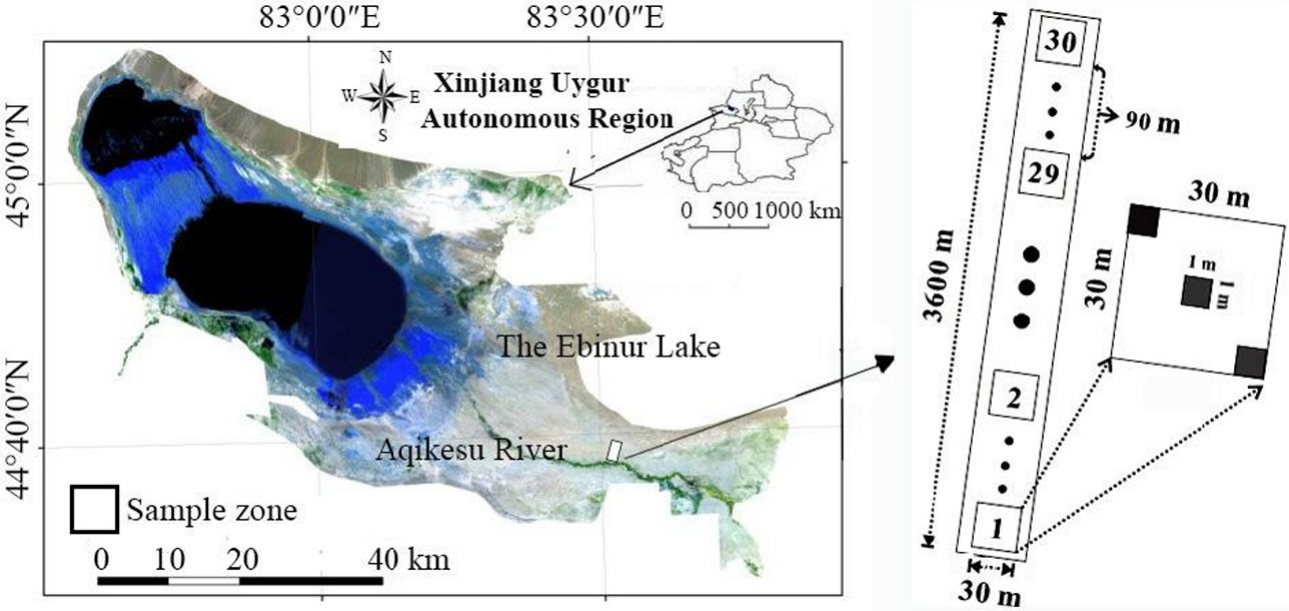
The study area and setup of the sample plots. Autonomous Region is downloaded from The Gateway to Astronaut Photography of Earth website (https://eol.jsc.nasa.gov/SearchPhotos/). Because the map downloaded from this website is free and open to scholars, our study does not need to supply a copyright notice).

A belt transect was established perpendicular to the Aqikesu River, The distance between East and West is 30 m, and that between North and south is 3600 m, in which thirty 30 m × 30 m plots were divided and separated by a distance of approximately 90 m. Three 1 m ×1 m herbaceous quadrats were set up in each 30 m × 30 m plot (Fig 1).

In each 30 m × 30 m plot, the arbor and shrub were investigated, and 1 m × 1m quadrats were used to investigate herbs. During the investigation, the species name, number of species, DBH, plant height and crown width of arbor plants were recorded, and the species name, number of species, height and coverage of shrubs and herbs were investigated. At the same time, the longitude and latitude of each sample were recorded.

Soil samples were collected using a diagonal sampling method in each 30 m × 30 m plot from three soil depth layers: 0–10 cm, 10–20 cm, and 20–30 cm. The collected soil sample from any given plot and soil layer was divided into two parts, with one part immediately placed into an aluminum box and the other into a self-sealing bag. The soil-filled aluminum boxes (weighed in advance) were numbered, and the fresh weights were recorded as soon as possible to determine the soil moisture content. The soil in the self-sealing bags was naturally dried to determine the soil physical and biochemical parameters. The soil moisture content was determined by drying the weighed samples at 105°C for 48 hours. The soil salt content was determined based on residue from an oven-drying method. Soil organic carbon was determined by a potassium dichromate volumetric-external heating method, the soil total nitrogen was measured using the Kjeldahl method [43], and the soil total phosphorus was determined by a perchloric acid-sulfuric acid digestion-molybdenum antimony anti-spectrophotometer colorimetric method [44].

### Statistical analysis

The β diversity index was calculated using the adiv package in R software [45]. The distribution of beta (hereafter β) diversity of different plant types was analyzed by a non-metric multidimensional scaling (NMDS) method. The relationships between the dissimilarity distance of species composition, geographic distance, and environmental distance were determined by a Mantel test and a partial Mantel test. Multivariate regression of the partial Mantel test was used to test the influence of spatial distance and environmental distance on the β diversity distribution pattern and was completed in the *phytools* package in R software. Redundancy analysis (RDA) was used to explore the relative contributions of environmental factors to desert plant community assembly. Variation partitioning analyses were conducted to determine the relative influence of environmental and spatial factors. NMDS, the Mantel test, the partial Mantel test, spatial autocorrelation, RDA, and variation partitioning were performed using the *vegan* package in R software [46–50]. The difference between the index of the community classification structure and the null model reflected the effects of environmental filtering and interspecific competition on community assembly [51].

## Results

### The variation pattern of β diversity in different plant types

**Fig 2:**
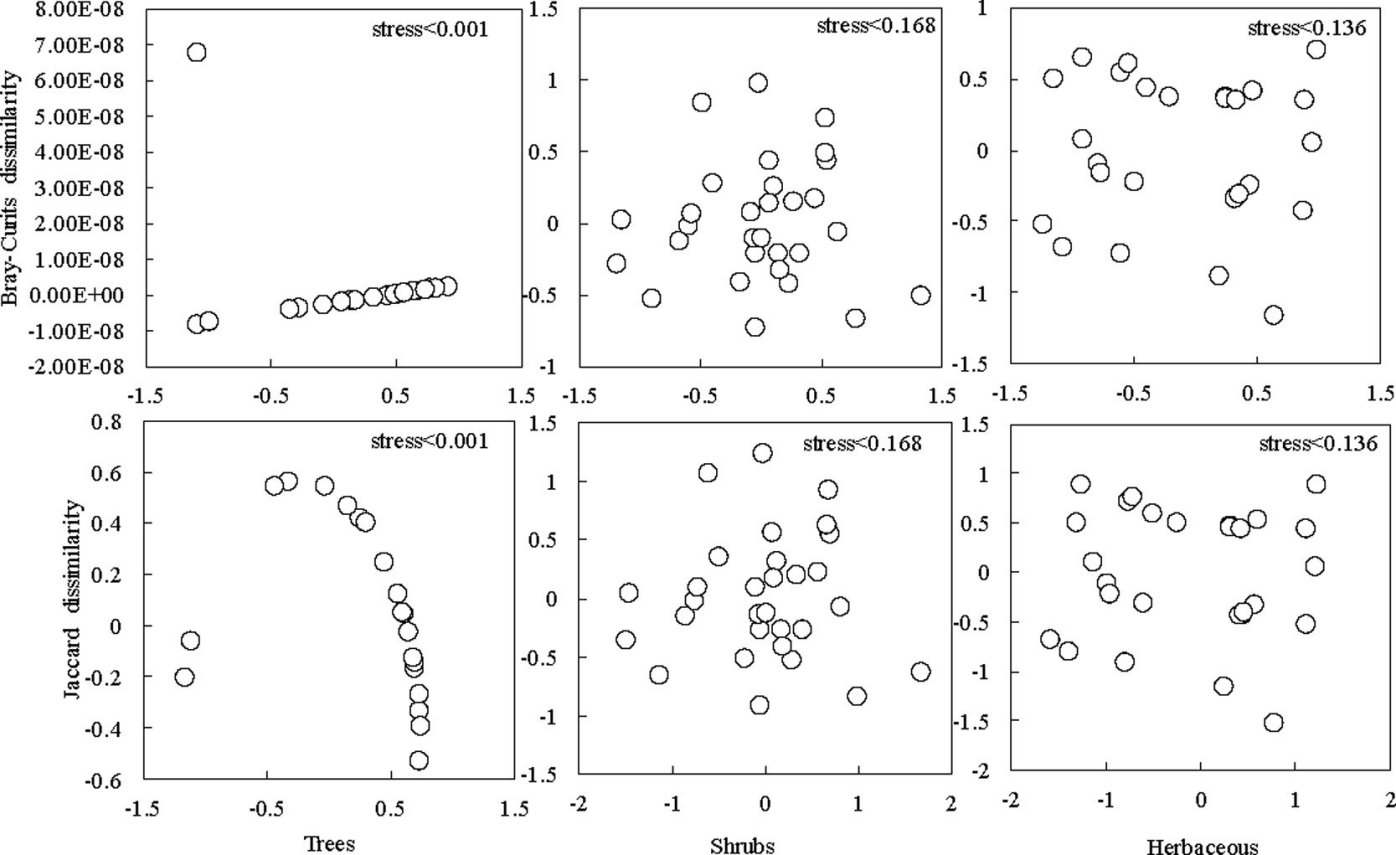
The changes in the beta diversity of different plant types.

The beta diversity (Jaccard dissimilarity) index of different life forms of plants was ranked in the order herbs (0.746) > shrubs (0.643) > trees (0.179). The stress values of the NMDS ranking results of different plant types were all less than 0.2 (Fig 2), indicating that the fitting results of the Bray–Curtis dissimilarity index and the Jaccard dissimilarity index of various layers of species passed the test. The distribution of tree species was usually relatively concentrated and overlapped, indicating that the species composition of the tree layer was relatively similar among samples. However, the herb and shrub layers were generally scattered with a low degree of overlap, suggesting that the species composition of the herb and shrub layers differed among samples.

**Fig 3:**
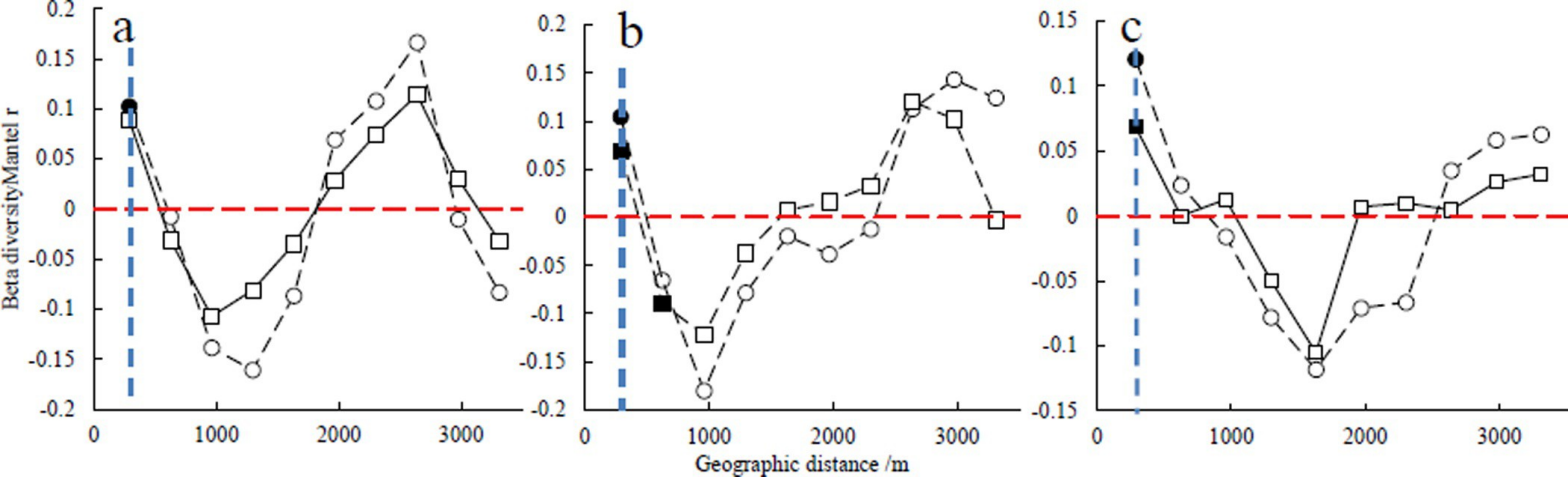
Spatial autocorrelation of beta diversity of different plant types: Trees (a), shrubs (b), and herbs (c). Note: The circles represent the spatial autocorrelation of β diversity, and the squares represent the spatial autocorrelation of β diversity after the addition of soil environmental factors. Solid circles or squares indicate significant spatial autocorrelation of β diversity (P < 0.05), while open circles or squares was not significant (P > 0.05). The blue vertical dotted line indicates a geographic distance of 288 m.

The spatial autocorrelation of β diversity of different plant types showed a fluctuating pattern with increasing distance. The spatial autocorrelation of β diversity of tree species presented an “inverted N” shape, while shrubs and herbs showed a “V” shape. The spatial autocorrelation of β diversity was significant at 288 m distance for tree, shrub, and herb species. After the addition of soil environmental variables, the spatial autocorrelation of the β diversity for the tree, shrub, and herb species decreased when the geographical distance exceeded 288 m, and the β diversity of the tree species did not show significant spatial autocorrelation (Fig 3).

### Correlations of community dissimilarity with geographical distance and environmental distance

The dissimilarity distances of tree, shrub, and herb species were significantly correlated with the environmental distance and geographical distance (*P* < 0.05). The correlation coefficients between species composition in the tree layer and the geographical distance and environmental distance were low: 0.149 and 0.104, respectively. Removal of the influence of geographical distance had almost no effect on the correlation coefficient between species composition and environmental distance. However, for the shrub and herb layers, the correlation coefficients between species composition and environmental distance were relatively high, i.e., 0.491 and 0.621, respectively. After removal of the effect of geographical distance, the correlation coefficients for the shrub and herb layers were 0.293 and 0.334, respectively. The correlation coefficients between the dissimilarity distance of species composition and the geographical distance were higher: 0.412 and 0.568 for the shrub and herb layers, respectively. After removal of the environmental distance, the correlation coefficients between the shrub and herb layers and the geographical distance were significantly smaller (Table 1).

**Table 1:** Mantel test and partial mantel test correlations for the community dissimilarity, geographical distance, and environmental dissimilarity of different plant types.

Items	Covariate	Trees	Shrubs	Herbs
		Mantel r	Mantel r	Mantel r
**Mantel test**				
Geographic distance	/	0.149*	0.412*	0.568*
Environment distance	/	0.104*	0.491*	0.621*
Water/salt	/	0.091	0.470*	0.690*
Carbon–nitrogen	/	0.133*	0.502*	0.563*
**Partial Mantel test**				
Geographic distance	Environment	0.104*	0.031	0.143*
Environmental distance	Geographic distance	-0.023	0.293*	0.334*
Water/salt	Geographic distance	0.056	0.252*	0.476*
Carbon–nitrogen	Geographic distance	0.021	0.316*	0.219*
Geographic distance	Water/salt	0.131*	0.053	0.002*
Geographic distance	Carbon–nitrogen	0.072	0.020	0.237*

Note: * represents a significant correlation of community dissimilarity with geographical distance or environmental distance. The confidence level is 0.05.

There were differential responses from different plant communities to soil water and salt contents. The correlation between the dissimilarity distance of the species composition of each layer in the community and the variation in the soil water and salt contents showed that the variations in the water and salt contents had no significant effect on the variation of species in the tree layer community, with a coefficient of 0.091 (*P* > 0.05) or even smaller, while the dissimilarity of the species composition of the shrub and herb layers had a stronger correlation with the variation in water and salt, i.e., 0.470 and 0.690, respectively (*P* < 0.05). After geographical distance removal, the correlation coefficients of water/salt and the dissimilarity of different types of plant communities were significantly reduced (Table 1).

The correlation between dissimilarity and carbon–nitrogen in different plant types was ordered as herbs (r = 0.563) > shrubs (r = 0.470) > trees (r = 0.133). After geographical distance removal, the correlation coefficients were 0.219, 0.316, and 0.021 for the herbs, shrubs, and trees, respectively.

### Comparison of the dissimilarity distance increase rates among different plant types

**Fig 4:**
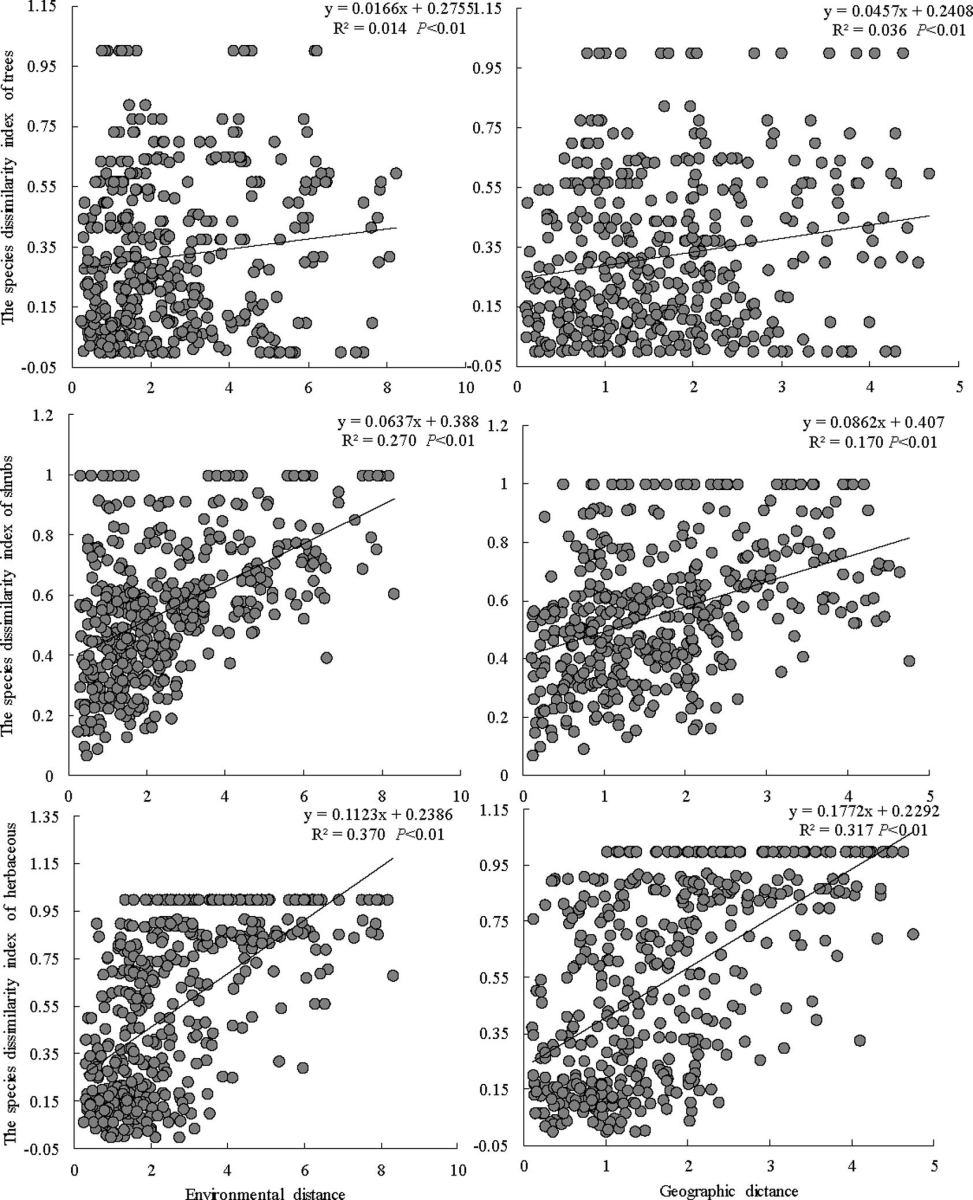
Relationships between the species diversity index and geographical distance and environmental distance.

**Fig 5:**
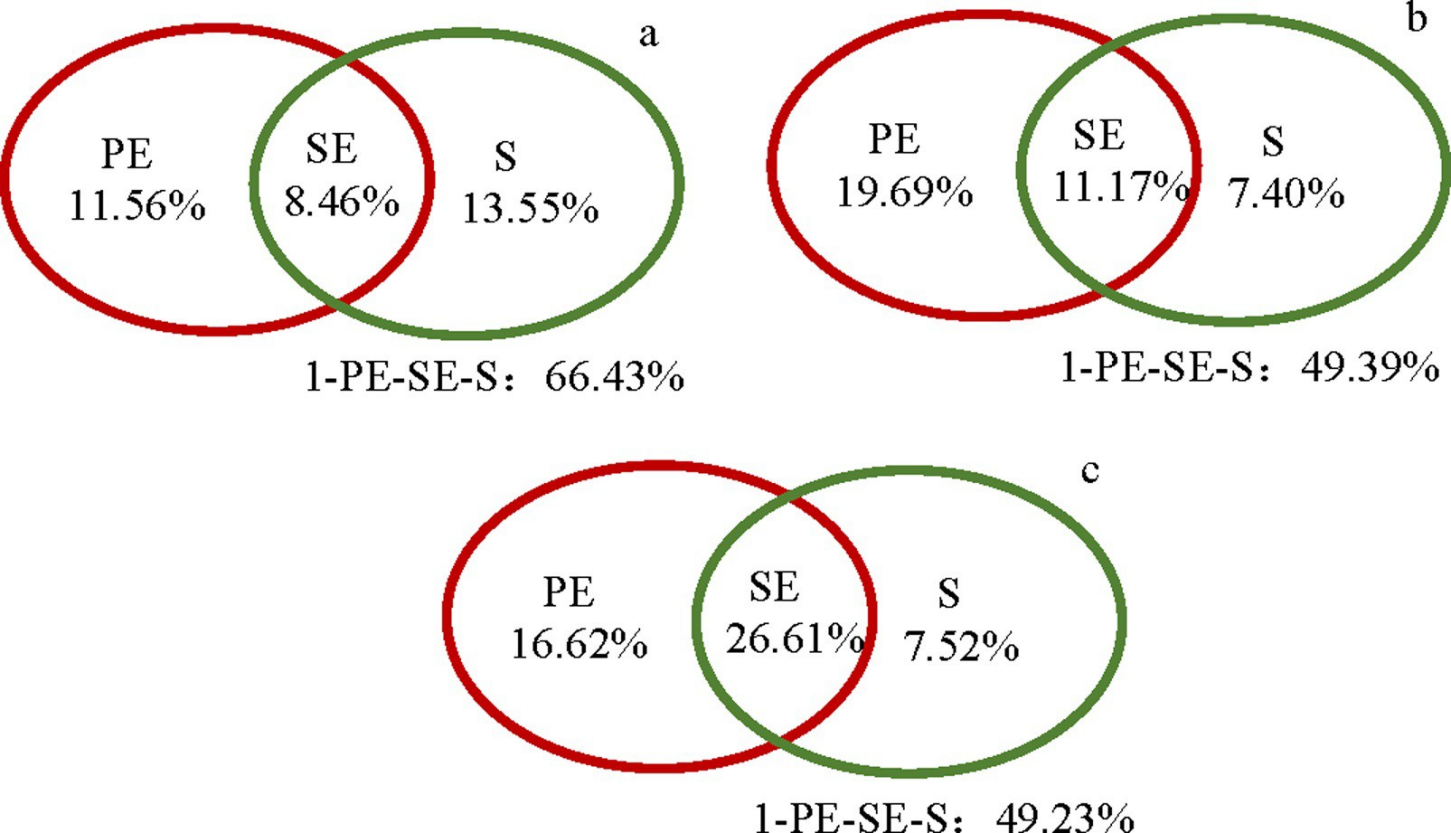
The explanation of environmental filtering and dispersal limitation for the desert plant community assembly. PE = pure environmental interpretation, S = pure spatial interpretation, SE = spatial environmental interpretation, and 1-PE-SE-S = unexplained variation.

With respect to the environmental distance, the relationship between the distance and the increase rates of dissimilarity of different plant types was herbs > shrubs > trees (Fig 5). The increase rates for herb and shrub species were 6.77 and 1.76 times higher, respectively, than that for tree species, and the difference between the rates for herbs and shrubs was extremely significant (*P* < 0.01). Along the geographical distance, the distance increase rate had an identical pattern (Fig 4), and the dissimilarity increase rate for herbs was 2.06 and 3.88 times higher than those of the shrubs and trees, respectively, and the difference was extremely significant (*P* < 0.01).

### Contributions of environmental and dispersal limitations to plant community assembly

A pure spatial interpretation metric (S) is often used to measure the relative size of dispersal limitation. Pure spatial variables explained 13.55%, 7.40%, and 7.52% of the variation in species composition for herbs, shrubs, and trees, respectively (Fig 5). Pure environmental variables significantly explained 16.62% and 19.69% of the species composition variation in the herb and shrub layers, respectively, but only explained 11.56% of the variation in the tree layer.

SE contributed 8.46%, 11.17%, and 26.61% of the variation in species composition in the herb, shrub, and tree layers, respectively; unexplained variation accounted for 66.43%, 49.39%, and 49.23% of the species composition variation in the herb, shrub, and tree layers, respectively.

### Relative contribution of environmental factors to desert plant community assembly

**Fig 6:**
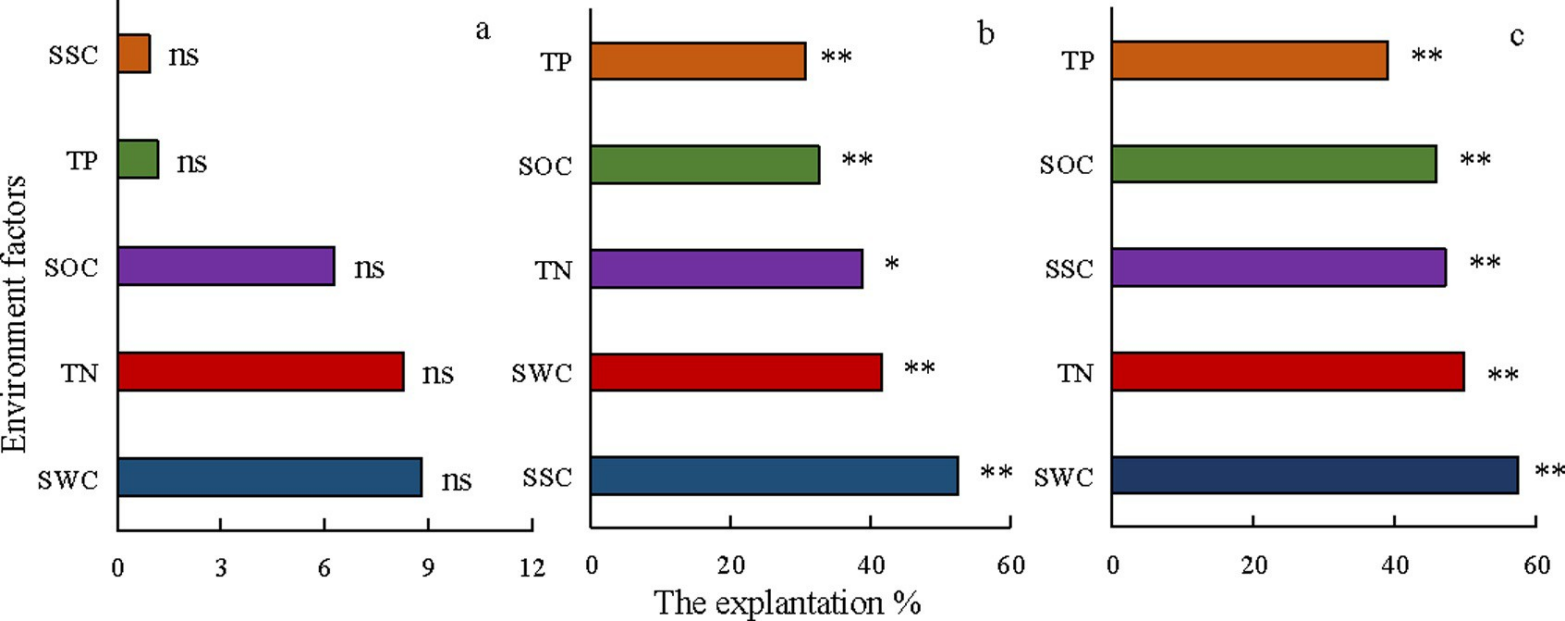
The relative contribution of environmental factors to the desert plant community assembly. Note: ns represents nonsignificant; * and * * represent significant and extremely significant effects of environmental factors on species composition at confidence levels of 0.05 and 0.01, respectively.

The RDA results showed that environmental factors had no significant influence on species composition variation in the tree layer (Fig 6). The order of importance of environmental factors on species composition of the tree layer was soil water content (8.8%) > soil total nitrogen (8.26%) > soil organic matter (6.28%) > soil total phosphorus (1.16%) > soil salt content (0.92%) (*P* > 0.05). Soil salinity was the most important influencing factor in the shrub layer (52.24%), while the minimum environmental factor was soil total phosphorus (30.78%), followed by soil water content (41.53%), soil total nitrogen (38.88%), and soil organic matter (32.68%). Soil water content was the optimal environmental factor affecting herb layer species composition, and the minimal factor was soil total phosphorus (39.07%).

## Discussion

As an important component of community species diversity, β diversity can reflect the degree of communication between species [52]. In this study, the β diversity of herbaceous plants was the highest, it may be because herbaceous plants have shorter growth cycle and faster regeneration rate [53]. These attributes allow herbaceous plants to quickly expand in suitable habitat areas, thus increasing species exchange in the region and thereby generating a high β diversity. However, due to the longer lifespan and slower rate of change of trees, the β diversity of trees was the lowest.

Environmental gradients and stochastic processes are two important driving forces for the formation of community spatial structure [12]. Environmental filtering refers to the effects of the environment on species without considering biological interactions, but biotic interactions may strongly shape ecological communities by affecting plant fitness, abundance, cover, and survival [17]. In order to distinguish between biological interaction and environmental filtering, standardized effect size (SES) values were used to compare the differences in taxonomic structure between 30 plots and their corresponding random communities. It was found that the SES values of the ratios of family richness to species richness (F/S) in 17 plots were negative, indicating that these communities were strongly affected by abiotic effects (such as environmental filtering), while other quadrats were strongly affected by competition (S1 Fig). However, the SES values of the ratios of generic richness to species richness (F/S) in most plots were positive, suggesting that competition had a greater influence on the ratio of genera to species. The effects of environmental filtering and competition on F/S and G/S were not consistent. This may be because an increase in environmental heterogeneity will weaken the impact of environmental filtering [54], thus increasing the opportunities for species of different genera or families to coexist. Another reason is that the intensity of competition and environmental filtration is related to the resources in the region where the species are located. When there are more resources, more individuals can be supported, which can reduce the competitive interactions among species require a similar resource [55]. Therefore, it is difficult to distinguish the effects of interspecific competition and abiotic filtering because the deviation of the taxonomic structure between field survey data and a null model likely results from the combined effects of both factors [56]. Therefore, this paper considered environmental filtering to explore the impact of niche process on species composition.

The relationships between the dissimilarity distance of species composition and geographical distance and environmental distance can be used to confirm the evidence for the existence of diffusion limitation and environmental filtering [7]. The variation in soil water and salt had no significant effect on the species changes in the tree layer. It is possible that trees, as plants with moderately deep and deep roots, play a role in hydraulic lifting that can transfer deep groundwater and soil water to dry surface soil and provide water for the roots of shallow plants, thus optimizing the root structure and showing better adaptability to the changes in water availability [57]. However, the effects of soil water and salt on the variation in herbaceous species composition were greater than that observed for the shrubs, which may be because shrubs are better able to use groundwater and river water than herbs, so shrubs can reduce the impact of soil moisture on shrubs composition by using both water sources. Our study suggests that the diversity of arid desert plants was affected by both diffusion limitation and environmental filtering.

The effect of soil C–N on tree and shrub β diversity was greater than those of water and salt, but the correlation coefficient between soil C–N and tree β diversity was low (Table 1). It has also been reported that soil nutrition had no effect on biodiversity [58]; however, this study area was in a typical desert with poor and arid soil, slow nutrient cycles, and low utilization rates [59]. Plant growth and distribution are generally limited by N in deserts [60]; however, due to the greater impacts of water and salt on plant distribution, the effects of N and phosphorus are often obscured. After the removal of the environmental distance or geographical distance, the correlation coefficients between the geographical distance or environmental distance and the dissimilarity of different plant communities decreased, indicating that the environmental distance was also affected by the spatial distance. Indeed, there was a significant positive correlation between the change in the environmental distance and spatial distance (S2 Fig). The differences in the correlations between species dissimilarity and soil C and N in different plant types communities may be due to the fact that the fertile island effect of tree species is greater than those of shrubs and herbs [61], and trees can resist wind and sand erosion and can intercept the fine-grained soil materials and dust around the tree crown. Most of the fine-grained soil materials eroded by the wind are in the surface soil, which contains relatively high amounts of organic matter and high C, N, and phosphorus contents, and thus there is a low correlation with the soil C and N contents outside the canopy.

Compared with plants in the shrub and herb layers, the increasing rate of distance of tree species dissimilarity was the lowest, indicating that trees were subjected to a higher degree of space restriction and that dispersal limitation played a significant role in tree species replacement [62]. The β diversity of herbs and shrubs varied with the geographical distance because herbs and shrubs are mostly shallow rooted or medium deep rooted plants, while trees are medium deep rooted plants, and the potential water sources and water use strategies of medium deep rooted desert plants are quite different. Trees mainly use groundwater, while shrubs mainly use river and soil water (S3 Fig), indicating a multifaceted strategy for the shrubs. For herbaceous plants, different from trees and shrubs, surface soil water is regarded as the primary water source and groundwater as the secondary water source. The overall performance was a relatively high average utilization rate of groundwater by herbaceous plants. Herbaceous plants and shrubs with medium and deep roots have similar water use strategies [63]. The diversity of water use leads to the greater variability of species composition.

As one of the main stochastic processes, species diffusion is related to the distance between communities. In recent years, geographic distance as a measurement of diffusion limitation has been widely used in the study of species spatial replacement [12, 22]. We found that the dissimilarity index of different types of plant species in the desert ecosystem increased with the increase in geographical distance, similar to previous research [22] that showed a decrease in the similarity index of species with the increase in geographical distance, or the dissimilarity index of species increased with the increase in geographical distance. This suggests that community composition changes with spatial distance could be due to ecological drift and limited transmission ability of the organism itself [64]. One research’s result show that the 100-seed weight of shrubs was greater than those of herbs and trees in desert ecosystem [65]. However, we found that the dispersal capacity of herbs was greater than that of shrubs and trees. This is inconsistent with the general finding that the larger the seeds, the more difficult the diffusion. Some studies have shown that seed quality is problematic in determining seed dispersal [66] due to the difficulty of dispersal for large seeds. The difficulty is often counter balanced by their advantages in seed release height, resource allocation, and dispersal mode [67]. There is a trade-off between time and space in seed dispersal. The trade-off between dormancy and dispersal is helpful to explain the greater dispersal distance of large seeds [68]. Species with larger seeds are expected to have lower dormancy rates, as their seedlings can take advantage of larger seed reserves and thus thrive in relatively adverse environments [69]. From this point of view, seed dormancy is determined by many factors, and there is a deviation from the seed quality.

Environmental filtering and dispersal limitation are not mutually exclusive in community assembly, but rather occur simultaneously [13]. Legendre et al. found that the relative contribution of each factor was related to the research scale and ecosystem type [70]. Compared with tropical forests, desert ecosystems in the Ebinur Lake Basin have more severe habitat conditions, and the composition of herbaceous and shrub species was shown to be affected by environmental filtering and dispersal limitation, with the niche process of environmental filtering playing a leading role. For species coexistence and biodiversity maintenance, in terms of dynamic and stable communities, neutral processes may be dominant in species-rich communities (such as tropical rainforests) [71], while in communities with relatively few species (such as temperate forests), the niche process may be dominant [72]. Although environmental filtering has a significantly higher contribution, one cannot exclude the importance of dispersal limitation, because the relative effect of separating the two depends on the quantity, quality, and spatial variables of the environment as well as the research methods used [22]. In addition, the relative contributions of dispersal limitation and environmental filtering also depend on the traits of species (e.g., the different plant types in this study) [73]. This study showed that the mechanism of species diversity and community assembly in the desert ecosystem was dispersal limitation in the tree layer (pure spatial variables had a higher contribution rate (13.55%) than that of environmental filtering (11.56%), while environmental filtering had a higher explanatory power for species composition variation in the shrub and herb layers. Our results showed that the process of community assembly of different types of plants was obviously different due to the differential importance of environmental filtering and dispersal limitation for different species groups with different diffusion abilities and habitat specialization [74]. Generally, environmental filtering has little effect on species with larger individuals [75] such as tree species, which may be due to the developed root system and sufficient light resources for those species. At the same time, the utilization efficiency of resources by larger species was higher than that of species with smaller individuals. In contrast, the assembly of communities with plants characterized by high diffusivity, rapid growth, and dormancy, such as shrubs and herbs, was mainly affected by the deterministic process of environmental filtering.

Environmental filtering is powerful in arid areas, which makes some desert plants adopt a variety of strategies to allow successful germination and seedling survival under limited water conditions. For example, the seeds of some desert plants produce mucilage, which can quickly absorb the scarce available water to ensure germination under harsh conditions [76]. Sand burial is an important environmental factor in sandy deserts [77]. Previous studies have shown that sand burial affects seed germination, seedling growth, plant growth, and community structure and function [78]. The possible reasons for moderate sand burial promoting seedling growth include an increase in the soil water content, space for the expansion of the root system, higher amounts of nutrients in the new sand area, and an improvement in the micro-environment [79]. In the occasional extreme drought years, when the rainfall amount or duration is insufficient for plant maturation or even seed germination, the short-term seed bank becomes the only means to ensure the sustainable survival of the population [80]. There are many Halophytes in desert area. The results showed that the germination patterns of halophytes are different; generally, most halophytes maintain seed vigor under long-term storage, thus increasing the possibility of successful seed recovery [81].

The physical and chemical properties of the soil can significantly affect the spatial changes in plant species composition, species structure, and ecosystem function [82]. At the local scale of arid ecosystems, soil characteristics affect plant distribution and coverage [83]. The soil water, salt, organic carbon, total nitrogen, and total phosphorus contents were significant environmental factors that affected the species composition of the herb and shrub layers, while these environmental factors had no significant effect on the tree layer. These factors may determine the species composition and changes in the community through resource constraints, regeneration constraints, and direct allelopathy [84]. The key environmental factor affecting the species distribution in the herbaceous layer was the soil water content, compared with the soil salt content in the shrub layer, which may be related to the ecological adaptability of plants to drought and salt stress. In habitats with high amounts of water and salt, salt-tolerant shrubs or herbaceous plants (such as *Suaeda salsa*, *Suaeda microphylla*, *Glycyrrhiza uralensis*, and *Halostachys caspica*) were reported to be evenly distributed, while in low-salt habitats with extremely limited soil moisture, only a small number of drought-tolerant herbs or shrubs were distributed, i.e., *Salsola ruthenica*, *Horaninowia ulicina*, *Calligonum mongolicum*, and *Agriophyllum squarrosum* [40]. Therefore, the increases in salt and drought stress may be the main reason for the significant change in the shrub and herb species composition, which again suggests that the limiting factors affecting the plant communities in this arid desert ecosystem were the water and salt contents [41].

Taxonomy-focused beta diversity may sometimes be insufficient. Thus, present-day research normally discusses the transformation of community assembly mechanisms from a single perspective (species diversity) to multiple dimensions (species, function, and phylogenetic diversity). One recent study showed that Spatial distance explained more variation in the taxonomic beta diversity of liana communities in a valley savanna; however, functional and phylogenetic beta diversity were more affected by environmental filtering [85]. In recent years, ecologists have studied community assembly from macroorganisms to microorganisms. Cao et al. [86] found that bacterial, fungal, and plant communities were strongly influenced by niche processes, i.e., environmental filtering, including soil and climate factors at the regional scale in arid and semi-arid areas. However, dispersal limitation was the main mechanism of fungal communities at the fine scale, whereas niche processes likely drove patterns of assembly at the regional scale in rubber plantations and rainforests [87]. In general, different dimensions, scales, and biological groups will be the focus in the study of community assembly mechanisms in the future.

## Conclusion

By analyzing the distribution patterns of β diversity of different plant types in the desert of Ebinur Lake Basin, we found that there were obvious differences in the characteristics of the β diversity of different plant types. The β diversity of herbs and shrubs was affected by both dispersal limitation and environmental filtering, and environmental filtering played a more important role. The change in tree species composition was significantly affected by spatial distance. The unexplained portion accounted for a large proportion of the variation. Some studies have shown that this is related to the selected quantity and the role of the microhabitat, factors that should be considered in future studies. Therefore, environmental heterogeneity and geographical difference should be simultaneously considered in the protection of plant diversity in desert ecosystems such as those of Ebinur Lake Basin.

## Supporting information

S1 Fig Standardized effect size (SES) of the null model for 30 plots in this research. (DOCX)

S2 Fig The regression relationship between environmental distance and spatial distance. (DOCX)

S3 Fig The utilization rates of different life forms of plants relative to each potential water source. (DOCX)

S1 Data (XLSX)
