# Supplementary material for: Characteristics and driving mechanisms of species beta diversity in desert plant communities
Source: PLoS One. 2021 Jan 11;16(1):e0245249. doi: 10.1371/journal.pone.0245249 (PMC7799812; doi:10.1371/journal.pone.0245249)
Supplement: S1 Fig — (DOCX) [file pone.0245249.s001.docx]

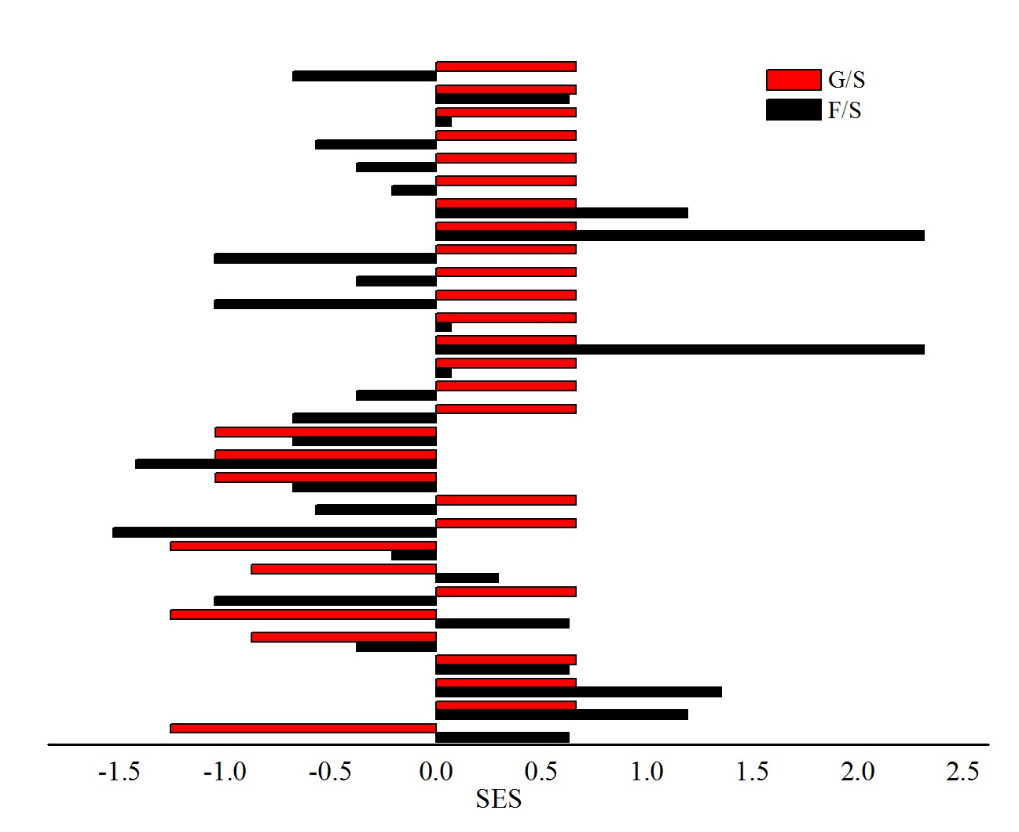


**S1 Fig Standardized effect size (SES) of the null model for 30 plots in this research**

Note: F/S represents the ratio of family richness relative to species richness, and G/S represents the ratio of generic richness relative to species richness.
