# Supplementary material for: Characteristics and driving mechanisms of species beta diversity in desert plant communities
Source: PLoS One. 2021 Jan 11;16(1):e0245249. doi: 10.1371/journal.pone.0245249 (PMC7799812; doi:10.1371/journal.pone.0245249)
Supplement: S2 Fig — (DOCX) [file pone.0245249.s002.docx]

**S2 Fig The regression relationship between environmental distance and spatial distance**
