# Supplementary material for: Characteristics and driving mechanisms of species beta diversity in desert plant communities
Source: PLoS One. 2021 Jan 11;16(1):e0245249. doi: 10.1371/journal.pone.0245249 (PMC7799812; doi:10.1371/journal.pone.0245249)
Supplement: S3 Fig — (DOCX) [file pone.0245249.s003.docx]

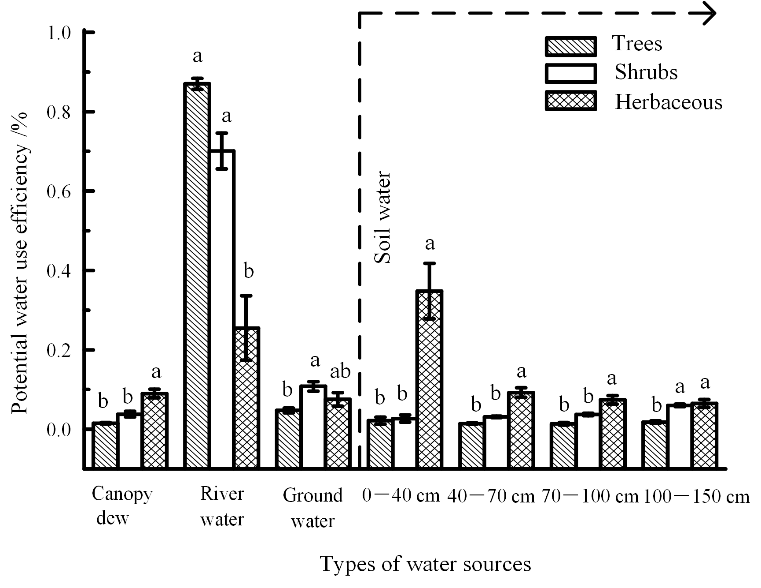


**S3 Fig The utilization rates of different life forms of plants relative to each potential water source**

Note: Different lowercase letters indicate significant differences among different plant types (*P* < 0.05), while the same lowercase letters indicate that there was no significant difference between different plant types (*P* > 0.05).
